# Increased Prevalence of Bisphosphonate-Related Osteonecrosis of the Jaw with Vitamin D Deficiency in Rats

**DOI:** 10.1002/jbmr.23

**Published:** 2010-01-14

**Authors:** Akishige Hokugo, Russell Christensen, Evelyn M Chung, Eric C Sung, Alan L Felsenfeld, James W Sayre, Neal Garrett, John S Adams, Ichiro Nishimura

**Affiliations:** 1 The Jane and Jerry Weintraub Center for Reconstructive Biotechnology, UCLA School of DentistryLos Angeles, CA, USA; 2 Section of Oral and Maxillofacial Pathology, UCLA School of DentistryLos Angeles, CA, USA; 3 Section of Hospital Dentistry, UCLA School of DentistryLos Angeles, CA, USA; 4 Section of Oral and Maxillofacial Surgery, UCLA School of DentistryLos Angeles, CA, USA; 5 Department of Biostatistics, UCLA School of Public HealthLos Angeles, CA, USA; 6 The Orthopedic Hospital Musculoskeletal Research Institute, The David Geffen School of Medicine at UCLALos Angeles, CA, USA

**Keywords:** osteonecrosis of the jaw, bisphosphonates, vitamin D deficiency, hyperparathyroidism, innate immunity

## Abstract

Necrotic bone exposure in the oral cavity has recently been reported in patients treated with nitrogen-containing bisphosphonates as part of their therapeutic regimen for multiple myeloma or metastatic cancers to bone. It has been postulated that systemic conditions associated with cancer patients combined with tooth extraction may increase the risk of osteonecrosis of the jaw (ONJ). The objective of this study was to establish an animal model of bisphosphonate-related ONJ by testing the combination of these risk factors. The generation of ONJ lesions in rats resembling human disease was achieved under the confluence of intravenous injection of zoledronate (ZOL; 35 µg/kg every 2 weeks), maxillary molar extraction, and vitamin D deficiency [VitD(−)]. The prevalence of ONJ in the VitD(−)/ZOL group was 66.7%, which was significantly higher (*p* < .05, Fisher exact test) than the control (0%), VitD(−) (0%), and ZOL alone (14.3%) groups. Similar to human patients, rat ONJ lesions prolonged the oral exposure of necrotic bone sequestra and were uniquely associated with pseudoepitheliomatous hyperplasia. The number of terminal deoxynucleotidyl transferase–mediated deoxyuridine triphosphate–biotin nick-end label–positive (TUNEL^+^) osteoclasts significantly increased on the surface of post–tooth extraction alveolar bone of the VitD(−)/ZOL group, where sustained inflammation was depicted by [^18^F]fluorodeoxyglucose micro-positron emission tomography (µPET). ONJ lesions were found to be associated with dense accumulation of mixed inflammatory/immune cells. These cells, composed of neutrophils and lymphocytes, appeared to juxtapose apoptotic osteoclasts. It is suggested that the pathophysiologic mechanism(s) underpinning ONJ may involve the interaction between bisphosphonates and compromised vitamin D functions in the realm of skeletal homeostasis and innate immunity. © 2010 American Society for Bone and Mineral Research.

## Introduction

Nitrogen-containing bisphosphonates (BPs), synthetic analogues of pyrophosphate, are effective in treatment of hypercalcemia of malignancy, osteolytic lesions in multiple myeloma, and bone metastases from solid tumors, including breast cancer and hormone-independent prostate cancer.(1) Serious complications are relatively rare (ranging from 0.1% to 1.8%) but include acute systemic inflammatory reaction, renal failure, and electrolyte imbalance.(2) However, a new concern is raised by the recent reports of osteonecrosis of the jaw (ONJ) in patients treated with these drugs.(3–5) Estimates of the cumulative incidence of ONJ from retrospective case reports and reviews(5–8) are as high as 10% among patients treated with BP intravenous infusions. The problem is not gender-specific and occurs in the mandible, maxilla, or both jaws.(5) In addition, oral BPs are currently the most widely used agents for the prevention and treatment of osteoporosis, although they are used at much lower bioavailable doses. While conclusive prevalence data in this patient group are not yet available, ONJ has begun to be recognized in a limited number of subjects receiving oral BPs.(9)

The American Society for Bone and Mineral Research (ASBMR) Task Force proposed the provisional case definition of ONJ as an area of exposed bone in the maxillofacial region that did not heal within 8 weeks in a patient who had been exposed to BPs.(10) ONJ typically is described as a failure of wound healing in the oral cavity or as severe, unresolved periodontal inflammation leading to necrotic bone exposure.(5,8,11) Tooth extraction is the highest risk factor for patients receiving BPs to develop ONJ. Kyrgidis and colleagues (2008) reported a case-control study in which tooth extraction during BP treatment significantly increased the risk (adjusted odds ratio of 16.4) of developing ONJ.(12) The ASBMR Task Force has further pointed out that there may be systemic risk factors related to cancer treatments.(10) It has been suggested that concurrent chemotherapy and dexamethasone may increase the risk of developing ONJ.(9) Sonis and colleagues (2009) reported ONJ-like lesions after tooth extraction in rats treated with zoledronate and dexamethasone.(13) However, the mechanism linking these risk factors to the development of ONJ has not been established.

Most ONJ lesions are observed in patients presenting a primary diagnosis of multiple myeloma or breast cancer.(5,9,14) A survey of multiple myeloma patients revealed that 40% had vitamin D deficiency (≤36 nmol/L) and 35% had vitamin D insufficiency (36 to 75 nmol/L).(15) Vitamin D insufficiency (<50 nmol/L) was reported for 30.2% of breast cancer patients.(16) Although the vitamin D status of ONJ patients has not been established,(17,18) the high-risk populations for ONJ appear to overlap with those with inadequate vitamin D levels. The implication of a role for vitamin D in ONJ is further seen in a prospective clinical study reporting that patients who exhibited significantly elevated serum levels of immunoreactive parathyroid hormone (iPTH) (likely owing to vitamin D insufficiency) prior to and throughout BP treatment eventually developed ONJ. Conversely, patients with normal iPTH levels prior to BP treatment did not develop ONJ.(17)

The objective of this study was to develop a rat model of ONJ that would consistently present lesions that are equivalent those seen in human disease. Because the pathologic mechanism of ONJ is virtually unknown, this study examined combinations of risk factors: BP intravenous injection, tooth extraction, and predisposing vitamin D deficiency. It was found that ONJ lesions developed most frequently in rats with all three risk factors.

## Materials and Methods

### Histopathologic characterization of human ONJ biopsy specimens

From the pathology archive of UCLA School of Dentistry from 2006 to 2007, 10 eligible microscopic slides were identified using the following criteria: confirmed clinical diagnosis of ONJ and recorded use of BP. The degree of histologic osteonecrosis was determined from the percent of empty osteocytic lacunae and pycnotic nuclei of osteocytes to the total number of osteocytes. In some cases, *Actinomyces* infection was evaluated by modified Grocott's methenamine silver (GMS) staining. This study was approved by the UCLA Institutional Review Board.

### Vitamin D–deficient rat model

In order to induce vitamin D deficiency [VitD(−)], 6- to 8-week-old Sprague-Dawley rats (Charles River Laboratories, Inc., Wilmington, MA, USA) were fed a diet lacking vitamin D (TD.89123: 0.47% calcium, 0.3% phosphorus; Harlan Teklad, Madison, WI, USA) and housed under a 12-h light/dark cycle. Ultraviolet B (UVB) emission (290 to 305 nm) in the action spectrum for cutaneous vitamin D synthesis was not detected from the fluorescent light fixtures in the animal housing. Vitamin D–sufficient rats were fed a normal rodent diet (TD.7013: 1.19% calcium, 0.92% phosphorus, 4.21 IU/g vitamin D_3_; Harlan Teklad, Madison, WI, USA) and kept in the same housing. Postfasting periphery blood was collected after 3 weeks. The serum samples were used to measure 25-hydroxyvitamin D_3_ [25(OH)D] and rat-specific immunoractive PTH (iPTH) by radioimmuno assays (Anilytics, Inc., Gaithersburg, MD, USA). The serum levels of calcium (Ca), phosphorous (P), alkaline phosphatase (ALP), and magnesium (Mg) were measured using a conventional method. C-terminal cross-linking telopeptide of type I collagen (CTX) and serum band 5 of tartrate-resistant acid phosphatase (TRACP-5b) were measured by enzyme-linked immunosorbent assays (RatLaps ELISA, Nordic Bioscience Diagnostics, Herlev, Denmark; RatTRAP Assay, IDS, Inc., Fountain Hills, AZ, USA, respectively). The data were analyzed using Student's *t* tests, and statistical significance was accepted for *p* < .05. All animal experiments were approved by the UCLA Animal Research Committee.

### BP intravenous injection

**Fig. 1:**
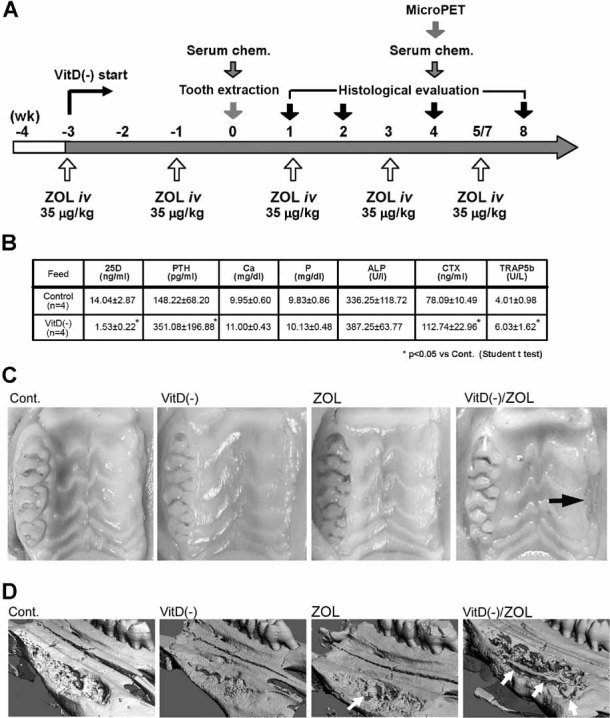
Rat ONJ model. (*A*) The treatment sequence to generate the confluence of factors: vitamin D deficiency [VitD(−)]; the serial intravenous injections of nitrogen-containing BP [zoledronate (ZOL)] and extraction of maxillary molar teeth. (*B*) Serum chemistry at the time of tooth extraction demonstrated the establishment of vitamin D deficiency. (*C*) Rat maxilla with tooth extraction wound healing (*right*) and untreated molars (*left*) harvested 2 weeks after tooth extraction. The VitD(−)/ZOL group exhibited delayed wound healing with the alveolar bone exposure (*arrow*). (*D*) Reconstructed µCT image of the rat maxilla 4 weeks after tooth extraction. The tooth extraction socket was nearly filled with newly formed bone. The wound healing of the VitD(−)/ZOL group appeared to be delayed, and detached bone sequestra of various sizes (*white arrows*) were observed.

The ZOL and VitD(−)/ZOL groups were produced by treating VitD(+) and VitD(−) rats with an intravenous injection of a bolus of zoledronate (Zometa, Novartis, Basel, Switzerland; 35 µg/kg in 0.9% NaCl) through a tail vein. The remaining rats, each in the control and VitD(−) groups, received an intravenous injection of vehicle solution (0.9% NaCl). The intravenous injection was repeated every 2 weeks (Fig. 1*A*). The human protocol for ZOL is 4 mg per infusion in intervals of every 3 to 4 weeks.(19) Given an average human body weight of 55 to 60 kg, the dose was determined as 70 µg/kg every 4 weeks. Because the excretion rate of injected ZOL in rats has not been established, we employed more frequent ZOL injections, 35 µg/kg ZOL every 2 weeks, but maintained the cumulative dose to be equivalent to the human dose of 70 µg/kg for the 4-week period.

### Tooth extraction

Maxillary left molar teeth were extracted from all groups at 3 weeks after initiating the systemic treatment using previously established methods.(20) Briefly, rats were anesthetized through inhalation of 2% isoflurane and placed in the supine position on a custom operation table. A dental explorer was used to separate the gingival attachment and to gently luxate the tooth. Subcutaneous injections of 0.05 mg/kg buprenorphine were given every 12 hours for 48 hours after tooth extraction, but no antibiotics were given.

### Identification of ONJ lesion and the prevalence of ONJ in rats

Then 1, 2, 4, and 8 weeks after tooth extraction, maxillae were harvested from the control, VitD(−), ZOL, and VitD(−)/ZOL groups. The tissue specimens were immediately fixed in 10% buffered formalin and photographed. The fixed tissue specimens were first examined by micro-computed tomography (µCT40, Scanco Medical, Bassersdorf, Switzerland) at an X-ray energy level of 55 kVp with a current of 72 mA. A 3D image of the maxilla was constructed using a customized computational program.

The tissue specimens then were decalcified with 10% ethylenediaminetetraacetic acid (EDTA) and processed for paraffin embedding. A series of 4-µm-thick sections in the frontal plane were prepared through the second to third molars of the intact side as the reference and in the same area around the extraction site. The number of total osteocyte lacunae and the number of empty osteocytic lacunae or pycnotic nuclei of osteocytes were counted within the buccual alveolar bone of the second molar for both the intact side and the contralateral (tooth extraction) side. In each section, the measurement was performed in five nonoverlapped fields under ×40 magnification. In the same specimen, when bone sequestra were observed, the number of total osteocyte lacunae and the number of empty osteocytic lacunae or pycnotic nuclei of osteocytes were counted separately. The ratio of nonvital osteocytes to the total osteocytes was calculated. Histologic bone exposure was determined by observation of direct communication to the oral cavity or oral epithelial tissue in approximation to the bone. The presence of *Actinomyces* infection was examined by GMS staining. The ONJ lesion was defined as alveolar bone or bone sequestrum containing areas of nonvital osteocytes with histologic bone exposure to the oral cavity.

The prevalence of ONJ was determined from specimens at 4 weeks after tooth extraction and was defined as the number of animals positive for histologic ONJ lesion(s) divided by the total number in the group and expressed as a percent. The statistical significance of ONJ prevalence between the experimental groups was determined by Fisher's exact test,(21) and statistical significance was accepted for *p* < .05.

### Serum chemistry and femur µCT bone morphometry

Peripheral whole-blood samples were collected 4 weeks after tooth extraction from the control, VitD(−), ZOL, and VitD(−)/ZOL groups (Fig. 1*A*), and serum chemistry was examined as disscussed earlier. Serum chemistry for each group was analyzed using one-way ANOVA followed by Dunnet's test, and statistical significance was accepted for *p* < .05.

Femur bones also were harvested and fixed in 10% buffered formalin and subjected to µCT scanning (µ-CT40, Scanco Medical, Bassersdorf, Switzerland) at an X-ray energy level of 55 kVp, a current at 72 mA, and an isotropic resolution of 16 µm. One-hundred and fifty slices (3.0 mm) were evaluated in the distal femur metaphysis at a threshold of 250.(22) The volume of interest included only secondary spongiosa. Trabecular bone morphometric measurements in 3D included bone volume normalized to tissue volume (BV/TV), trabecular structure connectivity density (Conn.D), trabecular number (Tb.N), trabecular thickness (Tb.Th), and trabecular separation (Tb.Sp). The data were evaluated by Dunnet's test, and statistical significance was accepted for *p* < .05.

### Characterization of osteoclasts in the maxillary alveolar bone

Histologic specimens of the maxilla surrounding the tooth extraction site at 4 weeks after tooth extraction were stained in situ for tartrate-resistant acid phosphatase (TRACP) using a commercially available kit following the manufacturer's protocol (Sigma, St. Louis, MO, USA). The number of osteoclasts defined by TRACP^+^ multinuclear cells (≥3 nuclei) was counted on the external surface of post–tooth extraction alveolar bone. Adjacent histologic sections were stained with in situ terminal deoxynucleotidyl transferase–mediated deoxyuridine triphosphate–biotin nick-end label (TUNEL) using a commercially available kit (Wako Pure Chemical Industries, Ltd., Osaka, Japan, and Calbiochem, San Diego, CA, USA). The number of TUNEL^+^ multinuclear cells (≥3 nuclei) on the external surface of post–tooth extraction alveolar bone was counted. The data were evaluated by Dunnet's test, and statistical significance was accepted for *p* < .05.

### Micro-positron emission tomography (µPET)

Four weeks after tooth extraction, static µPET images were obtained, using [^18^F]fluorodeoxyglucose (FGD) or [^18^F]fluoride ions freshly prepared by an RDS cyclotron (Siemens Preclinical Solutions, Munich, Germany). Rats (*n* = 4 in each group) were injected with 74 MBq of [^18^F]FDG or [^18^F]fluoride ion tracers via the tail vein and maintained under gas anesthesia for 1 hour, and then head scans were performed with a 10-minute acquisition time using µPET (FOCUS 220 system, CTI Concorde Microsystems LLC, Knoxville, TN, USA). Immediately afterward, non-contrast-enhanced µCT scans were obtained (µCAT II, Siemens Preclinical Solutions) with a 10-minute acquisition time. PET scan images were reconstructed using filtered backprojection and an iterative 3D maximum a posteriori (MAP) reconstruction technique.(23) Filtered backprojection images were used for quantification of tracer uptake by A Medical Image Data Examiner (AMIDE, Version 0.7.15, Geeknet, Inc., Mountain View, CA). Plain anteroposterior radiographs were superimposed on reconstructed PET images, and tooth extraction socket sites were identified and marked for each scan. Regions of interest (ROIs) in a box configuration measuring 2 × 5 mm then were drawn on horizontal images encompassing the extraction site. ROIs were applied to several successive planes measuring 3 mm in depth. The size of the ROI was standardized to approximate the actual size of the extraction wound. ROIs of identical size a were lso set at the contralateral nonextraction site as a control. Mean signal intensity (MBq/cc) within the volumes of interest was calculated using the AMIDE data-analysis tool. Values then were corrected for rat weight and actual tracer dose injected. Data were reported as standardized uptake value (SUV) and evaluated by two blinded reviewers. The percent of SUV was calculated to allow relative comparisons between the extracted and nonextracted sites. The data were analyzed by Dunnet's test, and statistical significance was accepted for *p* < .05.

### Expression of immune/inflammation-related genes

Total RNA samples were prepared from maxillary oral mucosa tissues overlying the week 4 tooth extraction site using a commercially available kit (Qiagen, Valencia, CA, USA). A quantitative real-time polymerase chain reaction (RT-PCR)–based array system (Rat Th1–Th2–Th3, PARN-034, SABioscience, Frederick, MD, USA) was used for assessing the expression profile of immune/inflammation-related genes. This array contained 84 spotted cytokine gene probes representative of helper T cells. In addition, RT-PCR of C—C motif chemokine 3 (*CCL3*; Rn00564660_m1, Applied Biosystems, Foster City, CA, USA) was performed. Total RNA from gingiva tissue harvested from intact maxilla of the control group was used as reference control.

## Results

### Human ONJ lesions

In the human ONJ biopsy specimens, the percentages of nonvital osteocytic lacunae were found to range from 66% to 100%, with an average 93% ± 12% (Table 1). GMS staining revealed *Actinomyces* colonies in bone marrow spaces or on the surface of sequestrum in 90% of specimens (Table 1). In 6 of 10 specimens, nonkeratinized epithelial tissue ingrowth into the lesions, so-called pseudoepitheliomatous hyperplasia (PEH), was observed adjacent to the bone sequestrum (Table 1).

**Table 1:** Histopathologic Evaluation of Human ONJ Biopsy Specimens

	Parameter		
	
Subject no.	*Actinomyces* infection	Pseudoepitheliomatous hyperplasia (PEH)	Empty osteocyte lacunae (%)
1	+	+	100.0
2	+	+	79.1
3	+	+	100.0
4	+	+	66.0
5	+	+	100.0
6	+	−	100.0
7	+	+	84.1
8	+	−	97.7
9	−	−	100.0
10	+	−	100.0
Summary	90%	60%	92.70 ± 12.08

### Establishment of vitamin D deficiency in rats

Rats fed a vitamin D–deficient diet for 3 weeks exhibited vitamin D deficiency. This was demonstrated by significant decrease in serum 25(OH)D compared with vitamin D–sufficient control animals (Fig. 1*B*). Serum calcium, phosphorus, and magnesium levels were not affected. In addition, iPTH CTX and TRACP-5b levels of the VitD(−) group were significantly increased over those of the control group. These results confirm the expected presence of secondary hyperparathyroidism and increased bone turnover in VitD(−) rats.

### Compromised tooth extraction wounding in the VitD(−)/ZOL group

Two weeks after unilateral maxillary molar extraction, the open wound of maxillary oral mucosa generally closed over the extraction site in the control, VitD(−), and ZOL groups. However, the VitD(−)/ZOL group showed delayed healing with alveolar bone exposure (Fig. 1*C*). The 3D reconstructed µCT images of the maxilla specimens 4 weeks after tooth extraction revealed that the extraction socket was filled with healing bone in the control, VitD(−), and ZOL groups. In contrast, the VitD(−)/ZOL group showed delayed and irregular wound healing and a number of bone fragments detached from the alveolar bone (*white arrows* in Fig. 1*D*). In all groups, no gross abnormalities were observed on the nonextraction side of maxilla with intact molar teeth and gingiva.

**Fig. 2:**
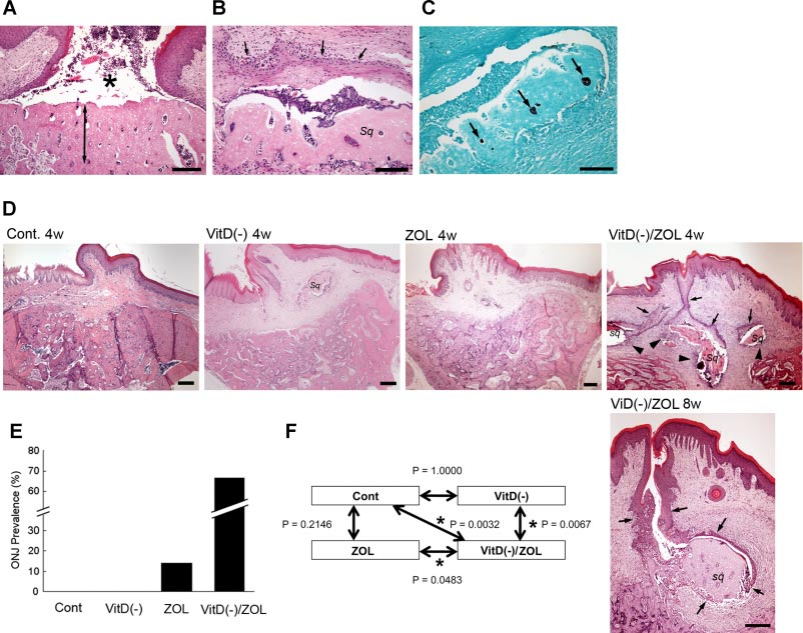
Histologic rat ONJ lesion. (*A*) In the VitD(−)/ZOL group, frontal sections through the second to third molars of rat maxilla 2 weeks after tooth extraction depicted partially necrotic alveolar bone (*arrow*) exposed to the oral cavity (*) through patent oral mucosa. Bar = 100 µm. (*B*) Detached necrotic bone sequestrum (*sq*) associated with pseudoepitheliomatous hyperplasia (PEH; *small arrows*) in a VitD(−)/ZOL rat. Bar = 100 µm. (*C*) Necrotic bone sequestrum in panel *B* stained with modified GMS indicated the presence of *Actinomyces*. Bar = 100 µm. (*D*) In the control, VitD(−), and ZOL groups, the tooth extraction socket was healed 4 weeks after tooth extraction. The sign of osteonecrosis was not observed. However, the necrotic bone sequestra (*sq*) sustained the oral exposure through PEH (*small arrows*) 4 and 8 weeks after tooth extraction in the VitD(−)/ZOL group. Bar = 200 µm. (*F*) The prevalence of rats exhibiting the osteonecrosis lesion defined as partial osteonecrosis with 60% or more empty osteocyte lacunae exposed to the oral cavity. (*G*) The prevalence in each group was compared by Fisher's exact test.

Two weeks after tooth extraction, the maxillary wound site of the VitD(−)/ZOL group revealed an area of nonvital alveolar bone (Fig. 2*A*). The wound edge of oral mucosa continued to be patent, and epithelial cells extended along the surface of necrotic bone. Along the deep margin of alveolar bone, there was immature bone with numerous cement lines associated with a diffuse, mild, inflammatory infiltrate. Four weeks after tooth extraction, partially necrotic bone fragments or sequestra (*sq* in Fig. 2*B*, Table 2) were observed, which were surrounded by inflammatory granulation tissue and were positive for *Actinomyces* infection (Fig. 2*C*). Although the wound edge of oral mucosa appeared to close, nonkeratinized oral epithelium grew toward the sequestrum, a lesion consistent with PEH (Fig. 2*B, D*). Necrotic bone sequestra with PEH seemed to persist in the VitD(−)/ZOL group even 8 weeks after tooth extraction (Fig. 2*D*). The oral exposure of bone sequestrum appeared to be established by PEH-mediated fistulation.

**Table 2:** Characterization of Maxillary Alveolar Bone Osteonecrosis

		Treatment			
		
Parameter		Control	VitD(−)	ZOL	VitD(−)/ZOL
Empty osteocyte lacunae of buccal alveolar bone (%)	*Unwounded side*	6.7 ± 1.5	6.2 ± 2.1	7.3 ± 3.2	7.4 ± 1.3
	*Tooth ext. side*	7.2 ± 1.2	6.9 ± 2.5	9.2 ± 4.9	11.3 ± 1.4 ***
Occurrence of bone exposure (%)	*Unwounded side*	0	0	0	0
	*Tooth ext. side*	0	0	14.3	66.7***
Number of bone sequestra after tooth extraction		0.3 ± 0.7	1.3 ± 1.8	0.9 ± 0.9	1.3 ± 1.5
Size of bone sequestrum (total osteocyte lacunae)		24.7 ± 24.5	50.1 ± 27.4	93.8 ± 16.8*	31.9 ± 22.0
Empty osteocyte lacunae/bone sequestrum (%)		54.8 ± 30.6	84.6 ± 14.2	83.7 ± 11.8	83.4 ± 14.3

* *p* < .5 versus control.

** *p* < .5 versus unwounded side.

In the control group, the tooth extraction wound showed active osteoclasts within resorption lacunae and lining osteoblastic cells compatible with repair of the maxillary defect (Fig. 2*D*, *Cont*). There were sporadic small epithelial inclusions surrounded by multinucleated giant cells (foreign-body type). Compared with the control group, the tooth extraction wound in the VitD(−) group was filled with more immature bone that contained numerous cement lines and osteoclasts and demonstrated extension of the hematopoietic marrow space to near the bone surface (Fig. 2*D*, *VitD(−)*). In the ZOL group, new bone in the extraction socket displayed prominent cement lines (Fig. 2*D*, *ZOL*). The intervening stroma was cellular and contained scattered inflammatory cells.

### The ONJ prevalence in rats

The histologic specimens taken 4 weeks after tooth extraction from each group were selected for the prevalence study. The ratio of nonvital osteocytes (necrotic bone area) was significantly greater in the buccal alveolar bone of the tooth extraction side than in the unwounded side (*p* < .05) in the VitD(−)/ZOL group. The necrotic bone area in the tooth extraction side was greater in the VitD(−)/ZOL group than in the control group (*p* < .05; Table 2). Bone sequestra were observed in all groups, although the number was highest in the VitD(−)/ZOL group. The size of bone sequestrum measured by the total number of osteocyte lacunae was found to be significantly larger in the ZOL group than in the other groups (Table 2). Bone sequestra of the ZOL group maintained the characteristic structure of alveolar bone, suggesting that traumatic bone fracture during tooth extraction might have occurred in some animals in the ZOL group. On the contrary, bone sequestra in the VitD(−) and VitD(−)/ZOL groups appeared to be subjected to extensive osteoclastic activity. Dense localized inflammatory cell infiltrates were associated with bone sequestra in the VitD(−)/ZOL group. However, inflammation was noticeably less severe in the VitD(−) group, in which bone sequestra were surrounded by granulation tissue (Fig. 2*D*).

Bone exposure to the oral cavity was observed most frequently in the tooth extraction side of the VitD(−)/ZOL group (Table 2). None of the unwounded side specimens indicated oral bone exposure. ONJ lesions were most prevalent in the VitD(−)/ZOL group (66.7%; four positive rats of six total rats), followed by the ZOL group (14.3%; one of seven). None of animals in the control (*n* = 9) and VitD(−) (*n* = 7) groups developed ONJ lesions. The Fisher exact test indicated that ONJ prevalence in the VitD(−)/ZOL group was significantly greater than in all other groups (*p* < .05; Fig. 2*F*).

### The effect of ZOL in serum chemistry and femur bone morphometry

The serum chemistry data (4 weeks after the tooth extraction) indicated vitamin D deficiency accompanied by hyperparathyroidism and hypocalcaemia in the VitD(−) and VitD(−)/ZOL groups (Table 3). The CTX level was elevated in the VitD(−) group, which was attenuated by ZOL treatment. The TRACP-5b level was decreased in the VitD(−)/ZOL group compared with the control and VitD(−) groups (Table 3).

**Table 3:** Serum Chemistry Measurements 4 Weeks After Tooth Extraction

	Parameter						
	
Treatment	25(OH)D (ng/mL)	PTH (pg/mL)	Ca (mg/dL)	P (mg/dL)	ALP (U/L)	CTX (ng/mL)	TRACP-5b (U/L)
Control	16.14 ± 2.91**	50.6 ± 7.2**	11.23 ± 0.17**	9.63 ± 1.20	172.7 ± 21.7	36.04 ± 4.97**	5.10 ± 0.81
VitD(−)	0.44 ± 0.06*	448.3 ± 27.2*	9.34 ± 0.74*	9.70 ± 0.31	270.2 ± 20.9	63.85 ± 5.93*	5.10 ± 1.29
ZOL	17.45 ± 1.63**	234.0 ± 62.2	10.42 ± 0.18	8.14 ± 0.25	212.4 ± 21.0	27.76 ± 3.54**	2.43 ± 0.32
VitD(−)/ZOL	0.62 ± 0.10*	649.2 ± 131.4*	7.46 ± 0.89*	9.62 0.45	276.8 ± 52.5	34.44 ± 3.46**	1.58 ± 0.16***

* *p* < .05 versus control.

** *p* < .05 versus VitD(−).

µCT bone morphometry of the femur metaphysis depicted osteopenic bone structure in the VitD(−) group, highlighted by decreased Tb.N and increased Tb.Sp. ZOL treatment not only reversed the VitD(−)-induced trabecular bone loss but further increased the bone structural indices (Table 4).

**Table 4:** Effect of VitD(−) and ZOL on Trabecular Bone Architecture as Assessed by µCT of the Femur Distal Metaphysis

	Parameter				
	
Treatment	BV/TV (%)	Conn.D. (mm^−3^)	Tb.N (mm^−1^)	Tb.Th (mm)	Tb.Sp (mm)
Control	18.70 ± 3.38	53.84 ± 11.92	2.89 ± 0.82**	86.00 ± 0.79	369.37 ± 119.99**
Vit D(−)	7.46 ± 2.19	26.53 ± 10.80	1.02 ± 0.23*	71.13 ± 3.56	1060.30 ± 268.73*
ZOL	57.92 ± 8.37***	163.95 ± 21.54*	7.50 ± 0.38***	129.00 ± 15.32***	115.78 ± 23.54**
VitD(−)/ZOL	41.54 ± 16.09***	170.32 ± 26.37***	6.39 ± 1.17***	97.78 ± 23.43	154.05 ± 46.26**

* *p* < .05 versus control.

** *p* < .05 versus VitD(−).

### Increased osteoclastogenesis and osteoclast apoptosis in the post–tooth extraction oral bone of the VitD(−)/ZOL group

**Fig. 3:**
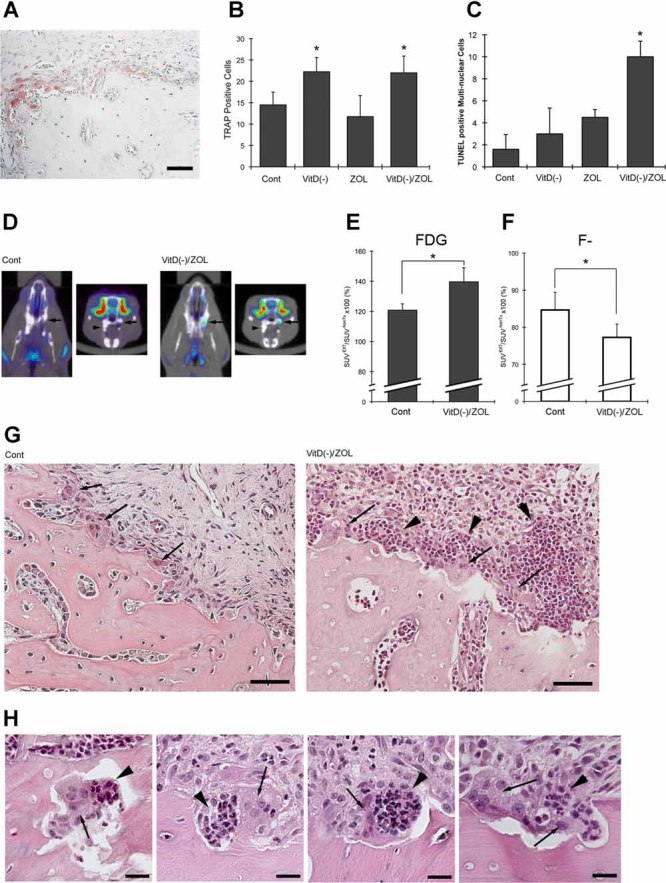
Characterization of the rat ONJ lesion. (*A*) A cluster of TRACP^+^ multinuclear cells at the external surface of alveolar bone at 4 weeks after tooth extraction in a VitD(−) rat. Bar = 50 µm. (*B*) The number of TRACP^+^ multinuclear cells (*n* = 4 in each group; ^*^p < .05 versus control). (*C*) The number of TUNEL^+^ multinuclear cells found for alveolar bone 4 weeks after tooth extraction (*n* = 4 in each group; ^*^p < .05 versus control). (*D*) Superimposed images of µPET with [^18^F]FDG and µCT. SUV of the tooth extraction site (*arrows*) was normalized, with the opposite side alveolar bone containing the remaining teeth (*arrowheads*) in the same animal. (*E*) The [^18^F]FDG SUV in the tooth extraction site normalized by the unwounded side (^*^p < .05 versus control). (*F*) The [^18^F]fluoride SUV in the tooth extraction site normalized by the unwounded side (^*^p < .05 versus control). (*G*) The surrounding oral mucosal tissue of post–tooth extraction alveolar bone of control and VitD(−)/ZOL rats. Osteoclasts (*arrows*) were found on the external surface. In the VitD(−)/ZOL group, a dense cluster of inflammatory cells (*arrowheads*) was found juxtaposed on the necrotic bone surface. (*H*) Osteoclasts (*arrows*) undergoing apoptosis of post–tooth extraction alveolar bone in the VitD(−)/ZOL group were associated with a cluster of inflammatory cells composed of neutrophils and lymphocytes.

The average number of osteoclasts (TRACP^+^ multinuclear cells) located on the external surface of post–tooth extraction alveolar bone (Fig. 3*A*) was significantly increased in the VitD(−) and VitD(−)/ZOL group compared with the control and ZOL groups (Fig. 3*B*). Following TUNEL staining, a larger number of TUNEL^+^ osteoclasts was observed in the VitD(−)/ZOL group compared with all other groups (Fig. 3*C*).

### Sustained inflammation in the post–tooth extraction oral bone of the VitD(−)/ZOL group

The metabolic activity of maxillary alveolar bone was evaluated by µPET. The standardized uptake value (SUV) of [^18^F]FGD for maxillary alveolar bone of the nonextraction side was evaluated initially by the two-way ANOVA, which indicated no significant difference among all groups (Fig. 3*D*). The normalized [^18^F]FGD SUV of the tooth extraction side was significantly higher in the VitD(−)/ZOL group than in the control group (Fig. 3*E*). The normalized [^18^F]FGD SUV of the tooth extraction side of the VitD(−) and ZOL groups did not differ significantly from control (data not shown). On the contrary, the normalized SUV of [^18^F]fluoride ion of the tooth extraction side decreased significantly in the VitD(−)/ZOL group compared with the control group (Fig. 3*F*), whereas other groups did not show any differences (data not shown).

In the VitD(−)/ZOL group, the localized inflammatory cell aggregation was observed adjacent to the bone surface, which was not seen in other groups (Fig. 3*G*). The densely localized inflammatory cells were composed primarily of neutrophils and lymphocytes. Uniquely, these inflammatory cells were found to juxtapose osteoclasts with pycnotic nuclei (Fig. 3*G, H*). There were no significant inflammatory cell infiltrates within the bone marrow space.

### Expression of immune/inflammation-related genes

**Fig. 4:**
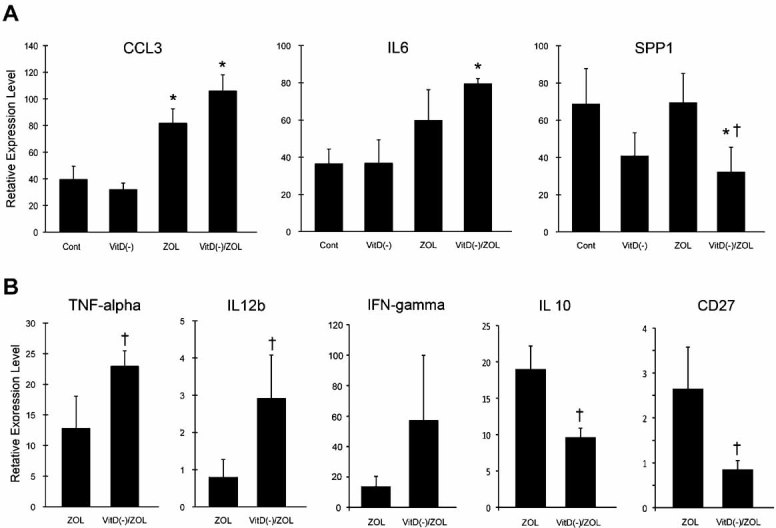
RT-PCR-based microarray assay depicted immune/inflammation-related gene expression. (*A*) The expression of *CCL3*, *IL6*, and *SPP1* in the oral mucosal tissues harvested from 4-week post–tooth extraction maxillae was affected by ZOL treatments but not by vitamin D deficiency alone. (*B*). In the ZOL-treated groups, vitamin D deficiency appeared to increase the expression of Th1-related genes (*TNFα*, *IL12b*, and *IFNγ*) and blunt the Th2-related genes (*IL10* and *CD27*) compared with the ZOL group. ^*^p < .05 versus the control group; †*p* < .05 versus the ZOL group.

The expression profile of Th1, Th2, and Th3 cytokine gene products was characterized in our model. Because apoptotic osteoclasts and accumulation of inflammatory cells were largely observed on the external surface of alveolar bone after tooth extraction, oral mucosa tissue overlying alveolar bone was selected for this experiment. Compared with the control group, the gene expression profile of the VitD(−) group was not significantly altered (Fig. 4*A*). The ZOL treatment groups showed elevated *CCL3*, *IL6*, and *IL6*. *SPP1* was found decreased in the VitD(−) and VitD(−)/ZOL groups, although statistical significance was reached only in the VitD(−)/ZOL group. Compared with the ZOL group, the VitD(−)/ZOL group had a clear tendency in elevated expression of Th1 cytokines such as tumor necrosis factor α (TNF-α) and interleukin 12b (IL-12b) (*p* < 0.05), as well as interferon-γ (IFN-γ) (*p* = 0.07). In contrast, Th2-related genes such as *IL10* and *CD27* were found to be downregulated in the VitD(−)/ZOL group (Fig. 4*B*).

## Discussion

### Establishment of a rat ONJ model

**Fig. 5:**
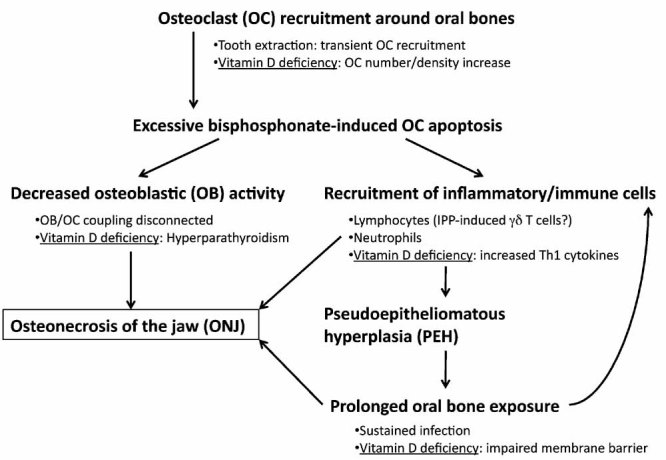
Hypothetical pathophysiologic mechanism of ONJ, in which the interaction between BP treatment and vitamin D deficiency may induce dysregulation of bone homeostasis and innate immunity.

This study demonstrated the development of ONJ lesions after tooth extraction in vitamin D–deficient rats treated with ZOL intravenous injections. Histologic characteristics of the rat ONJ lesion appeared to closely resemble the human disease. Human ONJ lesions have been shown to consist of multiple, partially confluent areas of necrotic bone containing residual nests of vital bone and were distinct from osteoradionecrosis lesions having extended homogeneous regions of complete bone necrosis.(24) The rat ONJ lesion similarly exhibited partially necrotic areas (Fig. 2*A, D*). The hallmark of confirmed ONJ is the prolong exposure of necrotic bone to the oral cavity.(10,25,26) Healing of the tooth extraction wound was delayed in the VitD(−)/ZOL group, where the oral wound did not close for at least 2 weeks. All other groups showed uneventful oral mucosa wound closure by gross observation(27,28) (Fig. 1*C*). Histologically, the exposure of necrotic bone to the oral cavity persisted at 4 and 8 weeks after tooth extraction in the VitD(−)/ZOL group. This exposure was indicated by food particles trapped between PEH and bone sequestrum (Fig. 2*D*). PEH was described originally as a skin disease with an exuberant proliferation of epidermal tissue(29) and has been reported as a rare complication of fistulated chronic osteomyelitis distinct from neoplastic lesions in the jawbone.(30) Akilov and colleagues (2007) demonstrated in the experimental cutaneous leishmaniasis mouse model that intralesional injections of TNF-α and IFN-γ initiated PEH in mouse ear skin.(31) The upregulation of TNF-α and IFN-γ in postextraction oral mucosa of the VitD(−)/ZOL group (Fig. 4) appears to provide a pathologic mechanism to develop PEH in our model. PEH was found in approximately 60% of human ONJ biopsy specimens(24) (Table 1). It must be noted that the unwounded side of alveolar bone did not show any sign of ONJ, suggesting that tooth extraction was a significant risk factor for developing ONJ in rats. From these findings, we propose that the persistent oral exposure of bone in ONJ may be mediated in part by PEH, which likely is induced by upregulated T-helper type 1 (Th1) cytokines associated with oral wounding (Fig. 5).

### The effect of ZOL

Our study reports the development of ONJ lesions in vitamin D–deficient rats receiving one or two human-equivalent parenteral doses. In this study, rats received two intravenous ZOL injections at the time of molar extraction (a cumulative total dose of 70 µg/kg) and received four injections (a cumulative total dose of 140 µg/kg) at 4 weeks after tooth extraction. However, the experimental dose of ZOL for rodents varies among reports. Croucher and colleagues (2003) used 120 µg/kg subcutaneous ZOL injections twice a week in a multiple myeloma mouse model (a cumulative dose of 240 µg/kg per week and 960 µg/kg for 4 weeks at the termination point).(32) Gasser and colleagues (2008) investigated the effectiveness of ZOL in a single intravenous injection in ovariectomized (OVX) rats with doses ranging from 0.8 to 500 µg/kg.(33) The attenuation of total bone mineral density (BMD) loss was found with as little as a single injection of 4 µg/kg of ZOL, and the effect of an injection of 20 µg/kg of ZOL was found to be equivalent to that of an injection of 200 µg/kg of alendronate. Treatment of female OVX rats(33,34) or male orchidectomized (ORX) rats(35) with various BPs showed the attenuation of bone morphometric measurements and decreased serum CTX or TRACP-5b values compared with untreated OVX or ORX rats. This study showed the effect of ZOL treatment on improvement of trabecular bone structure (Table 4). The elevated CTX values from VitD(−) treatment also were attenuated by ZOL treatment (Table 3). These data suggest that our experimental protocol generated the expected anticatabolic effect in bone.

BPs are known to be stable compounds and may be accumulated in skeletal tissues after repeated administration. It must be noted that BP treatment alone did not increase the prevalence of ONJ (Fig. 2), although the effect of long-term BP treatment was not addressed in our study. It may be possible that different doses as well as injection protocols could affect the prevalence and/or severity of ONJ. Further studies are needed.

### Apoptotic osteoclasts in the post–tooth extraction alveolar bone of the VitD(−)/ZOL group

Tooth extraction induces transient and localized osteoclastogenesis on the external surface of alveolar bone, which normally returns to the baseline level 4 weeks after tooth extraction in rats.(36) A novel observation in this study was that vitamin D deficiency appeared to sustain osteoclastogenesis even 4 weeks after tooth extraction (Fig. 3*A*). Anderson and colleagues (2009) reported the development of osteopenia in vitamin D–deficient rats associated with the increased osteoclast surface (Oc.S) in femurs and vertebral bones.(37) The increased ratio of steady-state mRNA levels of RANKL over osteoprotegerin (OPG) in femur bone marrow of vitamin D–deficient rats has been postulated to be responsible for the increased osteoclastogenesis.(37) In our study, the introduced vitamin D deficiency similarly may contribute to the increased osteoclastogenesis during tooth extraction wound healing.

TUNEL staining was found to be positive in approximately 10% of multinuclear cells in alveolar bone of the control and VitD(−) groups, whereas nearly 50% of osteoclasts in the ZOL and VitD(−)/ZOL groups appeared to become TUNEL^+^. As a result, a large number of osteoclasts underwent apoptosis in the VitD(−)/ZOL group (Fig. 3*C*). The combination of vitamin D deficiency and ZOL treatment may cause this extensive osteoclast apoptosis in the oral cavity.

### Decreased bone-formation activity of post–tooth extraction alveolar bone in the VitD(−)/ZOL group

µPET with [^18^F]fluoride ion depicts the de novo bone-formation activity. The noticeable decrease in uptake of [^18^F]fluoride ion to the tooth extraction wound of the maxilla of the VitD(−)/ZOL group may be due to the decreased bone turnover rate of jawbones (Fig. 3*D*). Vitamin D deficiency induced secondary hyperparathyroidism in rats (Fig. 1*B* and Table 3). Since osteodystrophy symptoms after renal failure have been linked to hyperparathyroidism,(38) the elevated PTH may have contributed to the decreased bone remodeling in the VitD(−)/ZOL group. However, because µPET with [^18^F]fluoride ion uptake was not modulated in the VitD(−) group with high PTH levels, its significance in the ONJ pathogenesis could not be determined in this study.

### Osteomyelitis-like inflammation and ONJ

µPET evaluations revealed that there was increased [^18^F]FDG tracer accumulation in the VitD(−)/ZOL group (Fig. 3*E*), indicating significant inflammation at the post–tooth extraction alveolar bone, similar to reports of human ONJ cases.(39) Wounding by tooth extraction induces transient inflammatory reactions, which generally subside by week 4 in rats.(40,41) The VitD(−)/ZOL group showed dense localized accumulation of inflammatory cells adjacent to necrotic bone composed largely of neutrophils and lymphocytes (Fig. 3*G, H*). Both [^18^F]FDG µPET and histologic features were thought to be consistent with osteomyelitis,(42) inflammation of bone and bone marrow owing to a pyogenic infection. In this study, rat ONJ lesions exhibited *Actinomyces* infection of a limited scope (Fig. 2*C*). Furthermore, the persistent communication to the oral cavity through PEH may make the bone sequestra in the VitD(−)/ZOL group susceptible to infection. Recently, the microbial-activated toll-like receptors have been shown to upregulate the expression of vitamin D receptors (VDRs) and CYP27b1-hydroxylase in human macrophages.(43) The human *cathelicidin* antimicrobial peptide gene is a direct transcriptional target of VDR-1,25(OH)_2_D interaction in those cells.(43,44) Therefore, an insufficient 25(OH)D level a may lso result in prolongation of bacterial infection of the mouth.

### Dysregulation of oral innate immunity as a potential pathophysiologic mechanism of ONJ

A unique histopathologic feature in the VitD(−)/ZOL group was that the dense inflammatory cell infiltration associated with the surface of partially necrotic bone, where a cluster of inflammatory cells was found juxtaposing apoptotic osteoclasts (Fig. 3*G, H*). This unique osteomyelitis-like inflammatory reaction may not be due only to bacterial infection. Nitrogen-containing BPs, once incorporated in the cell, interfere with the mevalonate biosynthetic pathway, in which BPs have been shown to compete with isopentenyl diphosphosphate (IPP) as a substrate of farnesyl diphosphate synthase (FPPS). As a result, IPP is abnormally accumulated, whereas downstream events of FPPS such as prenylation of small GTPase proteins are effectively prevented, and this leads to cell death.(47,48) It has been documented that the release of IPP from BP-treated monocytes serves as the putative ligand for T cells carrying γδ T cell receptor.(49) Although γδ T cells represent a small population of peripheral blood lymphocytes (1% to 5%), they can infiltrate into epithelial tissues, including oral mucosa.(50,51) It may be theorized that the excessive number of osteoclasts undergoing apoptosis in BP-treated groups could result in the release of IPP to the immediate field, above the critical level for activating γδ T cells in the oral mucosa. Because 1,25(OH)_2_D has been shown to inhibit phospholigand-induced γδ T cell expansion,(52) IPP-induced γδ T cells may further activated in the vitamin D–deficient environment. The IPP-induced γδ T cells have been shown to secrete the chemokine CCL3 (MIP-1α), a potent chemoattractant for neutrophil migration, at the equivalent level to those induced by bacterial lipopolysaccharides.(53,54) In this study, a significantly elevated CCL3 expression was found in the oral mucosa tissue of the ZOL and VitD(−)/ZOL groups (Fig. 4*A*), which may support the postulated role of BP-induced osteoclast apoptosis in the localized inflammatory reaction.

In contrast, Th1 cytokine expression pattern was different between the ZOL and VitD(−)/ZOL groups. Compared with the ZOL group, the VitD(−)/ZOL group had a clear tendency toward elevated expression of Th1 cytokines (Fig. 4*B*). Activated VDR- and CYP27B1 hydroxylase–expressing macrophages produce 1,25(OH)_2_D.(45) Acting through its receptor, 1,25(OH)_2_D is known to promote the innate immune response and limit the Th1 response in the local inflammatory microenvironment.(46) Our underlying hypothesis holds that diminished 1,25(OH)_2_D production in the oral mucosa in the presence of relatively low substrate 25(OH)D levels will impede the normal innate and adaptive immune reaction in response to massive BP-driven apoptosis of osteoclasts at the extraction site. Hence, in the absence of locally produced 1,25(OH)_2_D owing to diminished availability of substrate 25(OH)D, the Th1 response is amplified and the Th2 response is blunted. This was the case in the persistent wounds present in the vitamin D–deficient ZOL-treated rats reported here (Fig. 4*B*).

We did not observe a similar cytokine response from VitD(−) animals at 4 weeks after tooth extraction. We propose that this was the consequence of successful wound healing in that group in the absence of coadministered BP. We postulate that the interaction between BP treatment and vitamin D deficiency may generate a post–tooth extraction oral environment susceptible to neutrophil tissue injury(55) and major histocompatibility complex–unrestricted cytotoxicity by γδ T cells,(56) leading to localized osteonecrosis and prolonged exposure of the affected jawbone to the oral cavity through PEH (Fig. 5).

In conclusion, this article reports the successful development of ONJ lesions in rats under the confluence of three factors: (1] intravenous injections of ZOL, (2) tooth extraction wounding, and (3) predisposing vitamin D deficiency. We contend that our characterization of the disorder by gross anatomic observation and more precise lesional phenotyping by histology, µCT, and µPET provides convincing, clinically relevant evidence that the rat ONJ lesion closely mirrors human disease. We also posit that this rat model should be useful in dissecting the pathophysiologic mechanism(s) underpinning ONJ in humans. Furthermore, outcomes of our animal study suggest that patients susceptible to ONJ may be those with acquired or inherited disorders that compromise vitamin D synthesis, metabolism, or action in the realm of skeletal homeostasis and innate immunity.
